# Did the Paycheck Protection Program and Economic Injury Disaster Loan Program get disbursed to minority communities in the early stages of COVID-19?

**DOI:** 10.1007/s11187-021-00501-9

**Published:** 2021-05-05

**Authors:** Robert Fairlie, Frank M. Fossen

**Affiliations:** 1 Department of Economics, University of California, Santa Cruz, CA USA; 2 Stanford University, Stanford, CA USA; 3 NBER, Cambridge, MA USA; 4 Department of Economics, University of Nevada, Reno, NV USA

**Keywords:** Small business, Entrepreneurship, Business owners, Self-employment, Paycheck Protection Program, PPP, Economic Injury Disaster Loans, EIDL, COVID-19, Coronavirus, Shelter in place restrictions, Social distancing restrictions, Minority business, Racial inequality, J15, L26

## Abstract

Social distancing restrictions and health- and economic-driven demand shifts from COVID-19 shut down many small businesses with especially negative impacts on minority owners. Is there evidence that the unprecedented federal government response to help small businesses—the Paycheck Protection Program (PPP) and the related COVID-19 Economic Injury Disaster Loans (EIDL)—which had a stated goal of helping disadvantaged groups, was disbursed evenly to minority communities? In this descriptive research note, we provide the first detailed analysis of how the 2020 PPP and EIDL funds were disbursed across minority communities in the country. From our analysis of data on the universe of loans from these programs and administrative data on employer firms, we generally find a slightly positive relationship between PPP loan receipt per business and the minority share of the population or businesses, although funds flowed to minority communities later than to communities with lower minority shares. PPP loan amounts per employee, however, are negatively related to the minority share of the population. The EIDL program, in contrast, both in numbers per business and amounts per employee, was distributed positively to minority communities.

## Introduction

The widespread closing of stores and businesses in the USA and around the world due to the coronavirus is unprecedented. Stores, factories, and many other businesses have closed by policy mandate, downward demand shifts, health concerns, or other factors. The number of working business owners in the USA plummeted from 15.0 million in February 2020 to 11.7 million in April 2020 and has only partially rebounded since then (Fairlie, 2020).^1^ The impacts have also been disproportionately felt by race: business owner activity fell in the early stages of the pandemic by 41% among African-Americans and 32% among Latinx compared with 17% among whites.^2^

Given the impact of the pandemic, the federal government provided a response of larger magnitude than ever seen before in terms of providing financial assistance to small businesses. The largest program providing funds to small businesses is the Paycheck Protection Program (PPP) with a volume of $650 billion in the first two rounds during the early stages of the pandemic.^3^ The Small Business Administration (SBA)-administered program provides loans to small businesses through banks, credit unions, and other financial institutions with the stated goal of keeping small businesses open and retaining employees on the payroll. Loan amounts were generally equal to 2.5 months of average payroll costs, and can be forgiven if the business retains its employees. The program started providing loans on April 3, 2020, which was after most states imposed social distancing restrictions in response to COVID-19, and ran through August 8, 2020, providing more than 5 million total loans.^4^ The Economic Injury Disaster Loan Program (EIDL) program, which is also administered by the SBA, is designed to provide either loans or advances to small businesses that are losing revenues and sales due to COVID-19. Nearly 3.6 EIDL loans for $200 billion and nearly 5.8 million EIDL advances for $20 billion were provided to small businesses in 2020. EIDL loans can be used to cover up to 6 months of a wide array of working capital and normal operating expenses, such as continuation of health care benefits, rent, utilities, and fixed debt payments. EIDL advances are grants and do not have to be repaid, but are for smaller amounts ($1,000 per employee up to a maximum of $10,000). EIDL advances are subtracted from the forgiveness amount of their PPP loan if they are received in addition to PPP loans.

One of the stated goals in the CARES Act which included the PPP and EIDL programs was to prioritize serving “underserved markets” and businesses owned by “socially and economically disadvantaged individuals” (U.S. Congress 2020). Did the PPP and EIDL programs, which provided 15 million loans or advances worth more than $850 billion to small businesses, get disbursed to minority communities benefitting the businesses and employees in those communities? Given the larger negative effects of COVID-19 on business inactivity among minority businesses (Fairlie, 2020), targeting these relief funds to minority communities might be especially important.

In this descriptive research note, we provide the first detailed analysis of how the PPP and EIDL funds were disbursed across minority communities in the USA. Using administrative data on the universe of PPP loans, EIDL loans, and EIDL advances in 2020, we explore whether loans and advances were evenly distributed or not. We find that minority communities received a large share of PPP loans. We generally find a slightly positive relationship between PPP loan receipt per business and the minority share of the population. There is some evidence, however, that the first round of funds was disproportionately disbursed to non-minority communities and the second round of funds was disproportionately disbursed to minority communities. When we focus on the minority share of employer businesses in an area, we find similar results. Focusing on PPP loan amount per employee, however, we find a negative relationship with minority share of the population. In contrast, EIDL loans and advances, in both numbers per business and amounts per employee, were provided positively to minority communities. We find a strong positive relationship in the receipt per business of these loans and advances by minority share of the population.

These results build on the findings from a few related working papers on the PPP program. Granja et al. (2020) find that the first round of PPP funds flowed to areas more adversely affected by the economic effects of the pandemic, but that the early PPP did not have a substantial effect on local economic outcomes. Neilson et al. (2020) report based on survey data that the smallest businesses were less aware of the PPP, less likely to apply, and conditional on applications filed later, faced longer processing times, and were denied more often. Bartik et al. (2020) using firm-level data and an instrumental variables approach find that PPP loans led to an increase in a business’ expected survival, and a positive but imprecise effect on employment.^5^ Focusing on race, Lederer et al. (2020) conducted matched-pair audit testing of financial institutions in Washington, D.C. for PPP loans and find disparities between black and white testers in encouragement in applying for a loan, products offered, and information provided by the bank representative. Additionally, Erel and Liebersohn (2020) find that FinTech is disproportionately used to disburse PPP funds in high minority share ZIP codes. This paper builds on the previous research by providing the first comprehensive analysis of the relative disbursement of PPP and EIDL small business funds to minority communities, and the first study, to our knowledge, of the EIDL program. The findings are potentially important for future targeting and oversight of government aid to preserve minority businesses and the jobs they create.^6^

## Data

Partly in response to a Freedom of Information Act (FOIA) request and law suit threat by the media, the SBA released complete loan-level microdata for the PPP and EIDL programs. The PPP data cover the universe of loans provided through the program, which was from April 3, 2020, to August 8, 2020. The PPP is divided into two rounds. The first round ran from April 3 to April 16 and consisted of $342 billion across about 1.6 million loans. The second round ran from April 27 to August 8 and included more than 3.5 million loans with a total value of roughly $180 billion. In total, there are 5.2 million loans and $522 billion.

The loan microdata include information on the amount of the loan for loans under $150,000. For larger loans, only ranges are reported ($150,000–350,000, $350,000–1 million, $1–2 million, $2–5 million, and $5–10 million). Geographical information down to the zip code is provided in the smaller loan data, whereas exact address and even the name of the business are included in the larger loan data. The data also include information on industry, business type, jobs retained self-reported by the business, and name of the lender.^7^ Information on the race, gender, and veteran status of the owner is incomplete. The application did not ask for demographic information on the owners (see U.S. Small Business Administration, 2020 for the application form) and relied on banks to report the information. The result is that only 10% of loans provide race information, and these are heavily concentrated among a few banks.

The SBA also released loan and advance data from the EIDL program. The EIDL program data are separated into the loans and advances. There were 3.6 million loans and 5.8 million advances administered through the program. As of September 14, 2020, $190 billion of the allocated $200 billion in loans have been handed out to small businesses. All of the $20 billion for EIDL advances has been provided to small businesses.

To normalize the number of PPP or EIDL loans by zip code, we calculate loans per employer business. We use data from County Business Patterns (CBP) on business establishments with employees. The data are provided by the US Census Bureau at the zip code level as well as other geographical levels. The CBP data on employer establishments do not include counts of farms and non-profits. We acquire farm data by zip code from the US Department of Agriculture’s National Agricultural Statistics Service (NASS). From the PPP loan data, we exclude non-profit businesses, which represent 3.5% of loans, businesses with a non-classifiable industry (1.7% of the loans), self-employed persons (4.5%), and independent contractors (3.0%).

To normalize loan amounts, we calculate average loan amounts per business employee in each zip code. CBP data also includes employment levels for employer business establishments down to the zip code level. The normalization adjusts for loan amount differences due to differences in employment size by location, which is the general basis for loan amounts. Because only ranges are reported for larger loans in the PPP administrative data, we use the midpoint of each range for each loan (e.g., we use $250,000 for a recorded range value of $150,000–350,000). Using alternative assumptions such as 1/3 the range instead of the midpoint does not change the relationship by minority share. EIDL loan and advance amounts are complete.

We compare these measures of loan receipt per employer business establishment and loan amounts per employee to data from the Census of Population on the minority share of the population across communities. We measure minority share of the population primarily by zip code but also by county. In addition to analyzing the relationship between PPP and EIDL loan receipt per business by the minority share of the population, we examine the relationship by minority share of employer businesses. We use data from the Annual Business Survey (ABS) on employer businesses at the county level to calculate the minority share of employer businesses in each location. Data from the ABS are not available at the zip code level.

Table 1 provides mean values (weighed by population and unweighted) for the main variables of interest. Across zip codes, the average number of PPP loans per employer establishment is 0.489. The average loan amount per employee (unconditional on receiving a loan) is $4,404. EIDL loan receipt and amounts are lower. EIDL advances went out to more firms but the amounts were much smaller than other funds. The minority share of the population across zip codes has a mean of 0.389 and the minority share of employer businesses has a lower mean of 0.180 reflecting substantially lower business ownership rates among minorities.

**Table 1:** Descriptive statistics

	Weighted	Unweighted	*N*
	Mean	Mean	
PPP loans per employer establishment	0.489	0.347	31,952
PPP average loan amount per employee	$4,404	$4,892	30,356
EIDL loans per employer establishment	0.256	0.154	31,952
EIDL average loan amount per employee	$1,515	$2,488	30,356
EIDL advances per employer establishment	0.577	0.335	31,952
EIDL average advance amount per employee	$192	$262	30,356
Minority share of the population	0.389	0.229	32,670
Minority share of the population (county)	0.389	0.235	3,142
Minority share of employer businesses (county)	0.180	0.116	1,031

Notes: The statistics are at the zip code level if not otherwise indicated. The weighted means are weighted by population. Areas with unobserved minority shares are excluded. The PPP average loan amount per employee excludes loans to agriculture due to a lack of data on farm employees.

## Results

### Regional patterns in PPP loans and EIDL

**Fig. 1:**
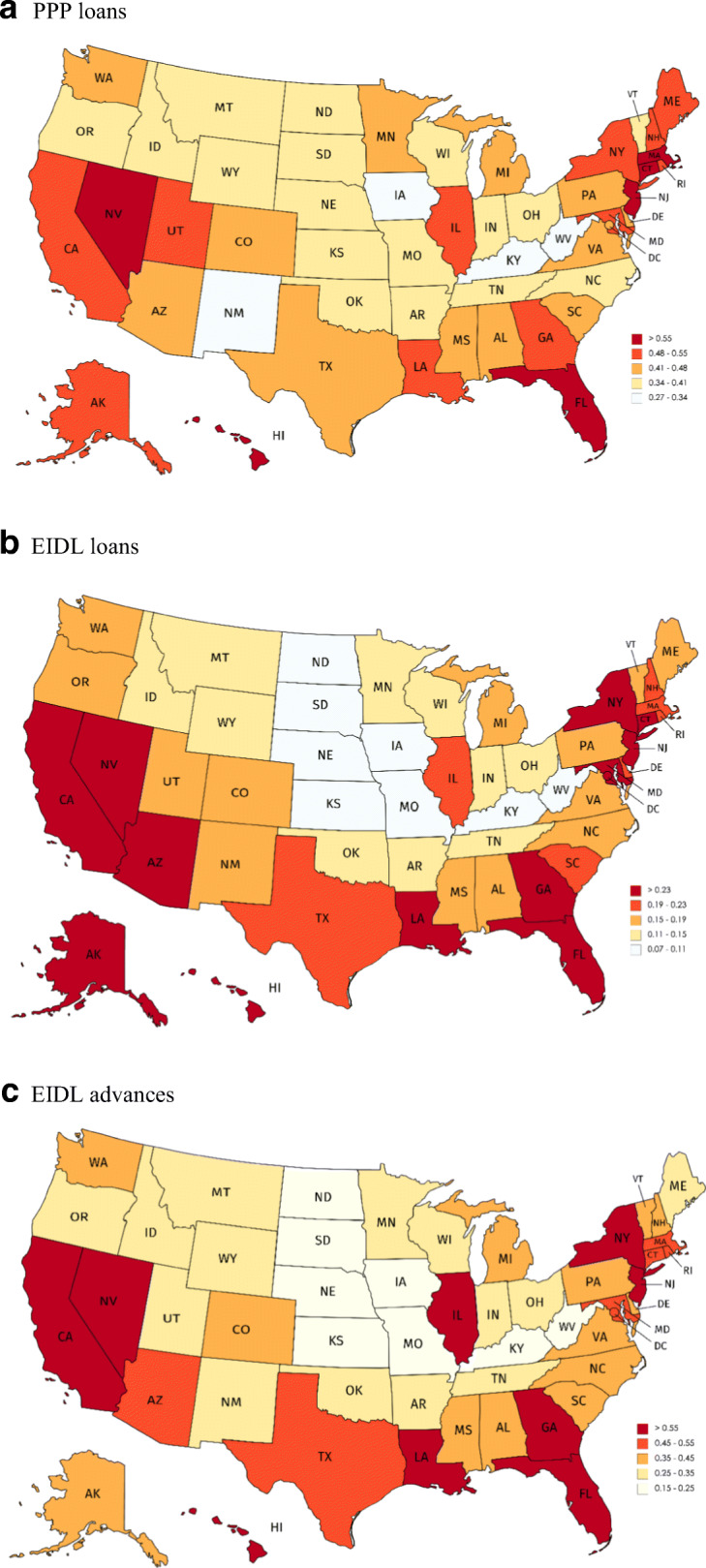
Distribution of loan receipts per employer establishment across states. Panel **a** PPP loans. Panel **b** EIDL loans. Panel **c** EIDL advances

PPP loans were spread across the country and not limited to a few regions. Figure 1, panel a, provides a state heat map of PPP loan receipt per employer business establishment. A few states had levels of above 0.55 loans per employer business and a few states had levels between 0.27 and 0.34 loans per employer business. States on the East Coast tended to have higher rates of loan receipt per business, and states in the Midwest tended to have lower rates of loan receipt per business. EIDL loan receipt per business (panel b) also was generally spread across the country. The patterns are somewhat stronger regionally, however, with the coasts having higher levels of loan receipt per business than the middle of the country. EIDL advances (panel c) show a somewhat similar pattern across states. The main takeaway from these figures, however, is that PPP loan, EIDL loan, and EIDL advances receipt per business was spread across the country and not limited to only a few states or regions.

### PPP loan receipt patterns by minority communities

**Fig. 2:**
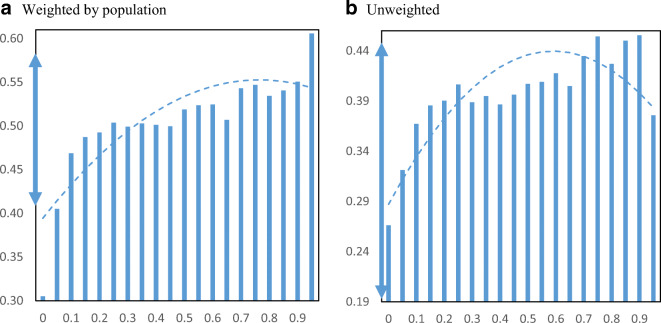
PPP loans per employer establishment by minority share. Panel **a** Weighted by population. Panel **b** Unweighted. **Notes**: The charts show the mean number of PPP loans per employer establishment in zip codes by minority share of the population. The dashed lines are from quadratic regressions at the zip code level. For perspective, the double arrow on the Y-axis indicates the median ± ½ standard deviation

We turn to analyzing how PPP loan receipt was distributed across minority communities. Figure 2 displays PPP loan receipt per employer establishment by minority share of the population at the zip code level. Panel a shows the relationship weighted by the total population and panel b shows the relationship without population weights. The figure also includes plotted quadratic regression lines to help show the relationship. Before discussing the results, two important points are noted. First, we do not report confidence intervals (i.e., “whiskers”) because we use the universe of PPP loans and administrative data on employer firms based on the Census Business Register. Second, we focus on the raw relationship between PPP loan receipt and minority share of the population without controlling for other factors because we are trying to capture the influences of these neighborhood characteristics. For example, if minority communities have higher poverty rates and that is correlated with receipt of PPP loans, then we want to include that in our measurement. Even if the driver of loan receipt is income, it is reflected in race and that is what we are trying to capture.

The relationship appears to be mostly flat between loan receipt and minority population share. Both weighted and unweighted figures show a slight positive relationship between loan receipt per business and the minority share of the population across zip codes in the USA. Most of the averages by minority share fall within the range of half a standard deviation from the median, as indicated by the double arrow on the Y-axis. Using the weighted figure, moving from the 25th percentile minority share of the population (16% minority) to the 75th percentile minority share of the population (59% minority) loan receipt only increases from 0.49 to 0.52 PPP loans per employer business establishment.

**Fig. 3:**
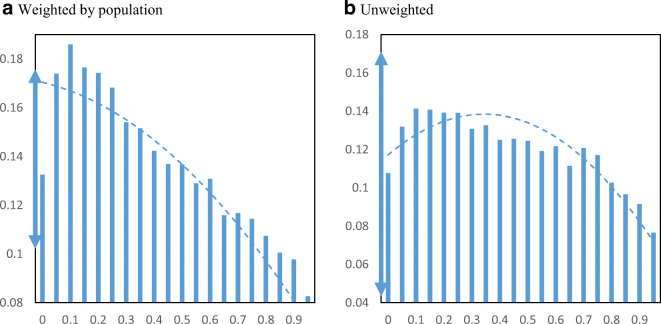
PPP loans per employer establishment by minority share in the 1st round. Panel **a** Weighted by population. Panel **b** Unweighted. **Notes**: The charts show the mean number of PPP loans per employer establishment in zip codes by minority share of the population in the first round of the PPP program (April 3–April 16, 2020). The dashed lines are from quadratic regressions at the zip code level. For perspective, the double arrow on the Y-axis indicates the median ± ½ standard deviation

**Fig. 4:**
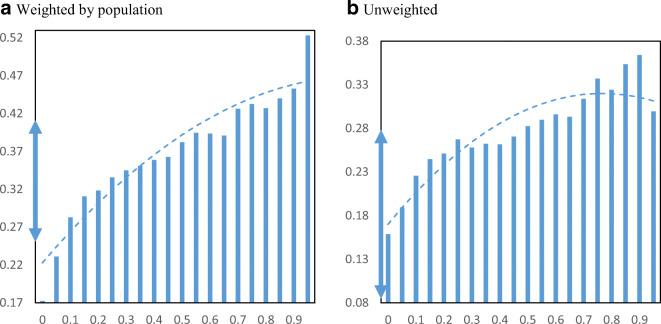
PPP loans per employer establishment by minority share in the 2nd round. Panel **a** Weighted by population. Panel **b** Unweighted. **Notes**: The charts show the mean number of PPP loans per employer establishment in zip codes by minority share of the population in the second round of the PPP program (April 27–August 8, 2020). The dashed lines are from quadratic regressions at the zip code level. For perspective, the double arrow on the Y-axis indicates the median ± ½ standard deviation

The PPP disbursed funds in two rounds with adjustments and awareness about the program changing between the two. The first round was April 3 to April 16, 2020, and consisted of 1.6 million loans. The second round ran from April 27 to August 8 and consisted of 3.6 million loans. Figure 3 displays the first round relationship, and Fig. 4 displays the second round relationship. Different patterns are revealed when separating by rounds. In the first round, loan receipt per business went disproportionately to non-minority communities. The figure shows a stronger negative relationship, with a decline of 0.05 loans per business between the 25th and 75th percentiles in minority shares. The second round of funding, however, showed the opposite pattern. In this case, there is an unequivocal positive relationship between loan receipt and minority population share. Moving from the first quartile to the third quartile in minority share is associated with an increase of 0.08 PPP loans per firm.

In terms of the different rounds of the PPP, the first $349 billion was exhausted after just 2 weeks of being available. Given unmet need by small businesses for assistance, Congress approved an additional $310 billion. The change in the slope of the relationship between the two rounds might be caused by a few factors. First, applying for PPP loans early on favored having long established relationships with banks which minority businesses were less likely to have (Mills & Battisto, 2020). Second, much of the early money flowed through smaller community banks which were often in rural areas because these banks were nimbler at accessing the aid (Bloomberg, 2020). In the second round, larger banks with more urban and racially diverse customer bases caught up. Third, minority-owned businesses tend to be smaller than non-minority-owned businesses (U.S. Census Bureau, 2016; Fairlie & Robb, 2008), and smaller businesses typically took longer to complete required paperwork because they often did not have in-house accountants, legal help, or other support. Finally, FinTech and other online lenders were brought in and approved by the SBA, and these lenders were often active in minority areas (Liu & Parilla, 2020). It is unclear how costly the delay was in receiving loans to minority businesses and communities.

**Fig. 5:**
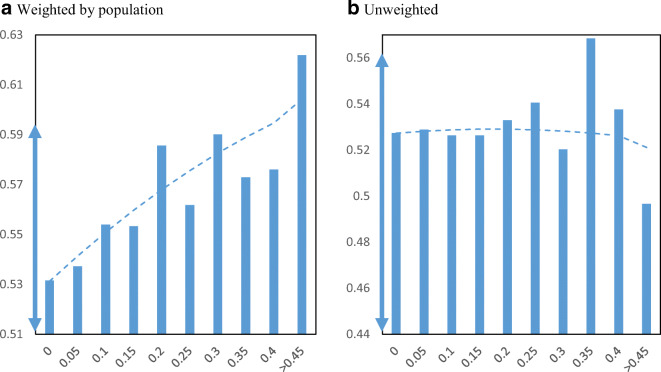
PPP loans per employer establishment by minority share of businesses. Panel **a** Weighted by population. Panel **b** Unweighted. Notes: The charts show the mean number of PPP loans per employer establishment in counties by minority share of businesses. Loans to agricultural businesses are excluded. The dashed lines are from quadratic regressions at the county level. For perspective, the double arrow on the Y-axis indicates the median ± ½ standard deviation

We turn to analyzing the relationship between PPP loans per business and the minority share of businesses in the community. To measure the minority share of businesses, we use data from the Annual Business Survey (ABS) on employer businesses at the county level.^8^ Data are not available at the zip code level.^9^ Figure 5 displays the relationship. The unweighted numbers do not indicate a clear pattern and are mostly consistent with a flat relationship. The weighted numbers by population size indicate a slight positive relationship. The relationship is not strong however. For example, moving from the 25th percentile of counties in the minority share of businesses (9% minority share) to the 75th percentile (25%) is associated with an increase of 0.02 PPP loans per employer business.

**Fig. 6:**
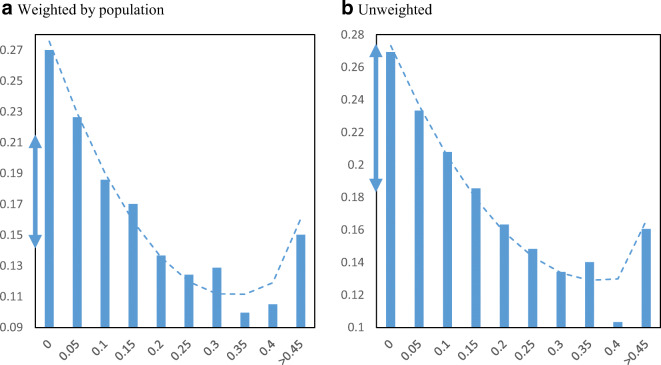
PPP loans per employer establishment by minority share of businesses in the 1st round. Panel **a** Weighted by population. Panel **b** Unweighted. **Notes**: The charts show the mean number of PPP loans per employer establishment in counties by minority share of businesses in the first round of the PPP program (April 3–April 16, 2020). Loans to agricultural businesses are excluded. The dashed lines are from quadratic regressions at the county level. For perspective, the double arrow on the Y-axis indicates the median ± ½ standard deviation

**Fig. 7:**
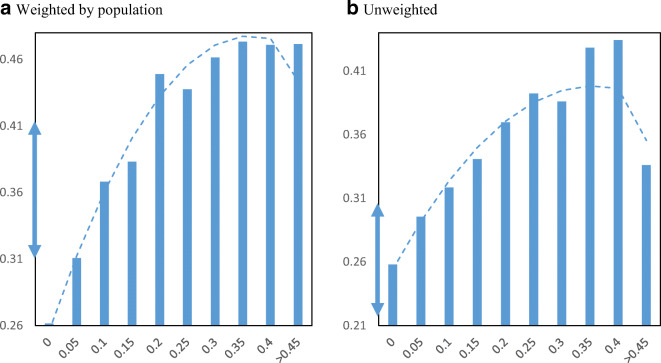
PPP loans per employer establishment by minority share of businesses in the 2nd round. Panel **a** Weighted by population. Panel **b** Unweighted. **Notes**: The charts show the mean number of PPP loans per employer establishment in counties by minority share of businesses in the second round of the PPP program (April 27–August 8, 2020). Loans to agricultural businesses are excluded. The dashed lines are from quadratic regressions at the county level. For perspective, the double arrow on the Y-axis indicates the median ± ½ standard deviation

Figures 6 and 7 display the relationship between loan receipt and minority business share for the first and second rounds, respectively. Similar to our findings using the minority share of the population, again we find that in the first round, there appears to be a negative relationship between loan receipt and minority business share, and in the second round, the relationship switches to being positive.

#### PPP loan amounts

**Fig. 8:**
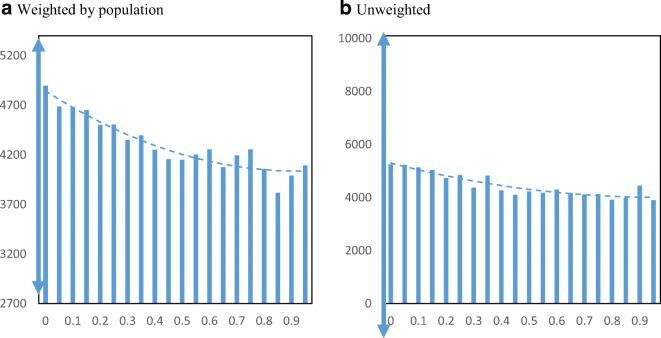
PPP loan amounts per employee by minority share. Panel **a** Weighted by population. Panel **b** Unweighted. **Notes**: The charts show the mean amounts of PPP loans per employee in zip codes by minority share of the population. Loans to agricultural businesses are excluded. Loans reported as a range are approximated by using the interval midpoint. The dashed lines are from quadratic regressions at the zip code level. For perspective, the double arrow on the Y-axis indicates the median ± ½ standard deviation

The disbursement of PPP funds across communities by minority share might differ when measured by loan amounts instead of number of loans. Figure 8 displays average loan amounts per business employee by minority share in the population at the zip code level. We standardize the Y-axis by reporting the range of ± ½ standard deviations around the median loan size (cutting off at zero in case of the unweighted chart). We find a slight downward relationship with minority share. Moving from the 25th to the 75th percentile in minority share is associated with a decrease from $4652 to $4204 in average loan amount per employee.

### Economic Injury Disaster Loan programs

Although the PPP program has received a lot of attention, the federal government also approved the $220 billion EIDL program, which also provides aid to small businesses during COVID-19, but has received much less attention. There are two programs, EIDL loans and EIDL advances. EIDL loans are not forgivable and must be paid back in full. EIDL advances are grants and do not have to be repaid, but are for smaller amounts ($1,000 per employee up to $10,000 total).

**Fig. 9:**
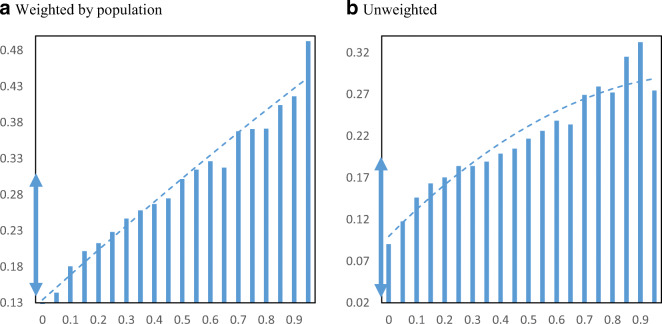
EIDL loans per employer establishment by minority share. Panel **a** Weighted by population. Panel **b** Unweighted. **Notes**: The charts show the mean number of EIDL loans per employer establishment in zip codes by minority share of the population. The dashed lines are from quadratic regressions at the zip code level. For perspective, the double arrow on the Y-axis indicates the median ± ½ standard deviation

Figure 9 displays EIDL loan receipt per employer establishment by minority share of the population across zip codes. The relationship between loan receipt and minority population share shows a clear upward pattern. If we move from the lowest quartile minority share (16%) to the highest quartile minority share (59%), loan receipt increases from 0.20 to 0.31 EIDL loans per employer business establishment.

**Fig. 10:**
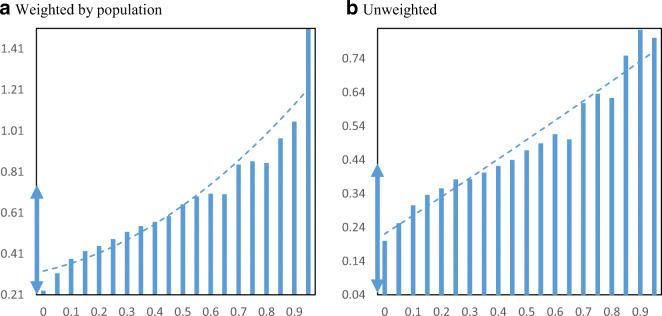
EIDL advances per employer establishment by minority share. Panel **a** Weighted by population. Panel **b** Unweighted. **Notes**: The charts show the mean number of EIDL advances per employer establishment in zip codes by minority share of the population. The dashed lines are from quadratic regressions at the zip code level. For perspective, the double arrow on the Y-axis indicates the median ± ½ standard deviation

Figure 10 displays EIDL advance receipt per employer establishment by minority share of the population in zip codes. The relationship between advance receipt and minority population share shows a similarly strong upward pattern. Movement from the lowest quartile to the highest quartile minority share loan receipt increases from 0.42 to 0.69 EIDL advances per employer business establishment.

#### EIDL loan amounts

**Fig. 11:**
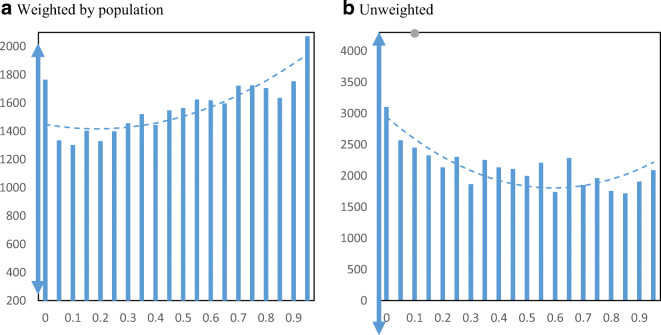
EIDL loan amounts per employee by minority share. Panel **a** Weighted by population. Panel **b** Unweighted. **Notes**: The charts show the mean EIDL loan amounts per employee in zip codes by minority share of the population. The dashed lines are from quadratic regressions at the zip code level. For perspective, the double arrow on the Y-axis indicates the median ± ½ standard deviation

**Fig. 12:**
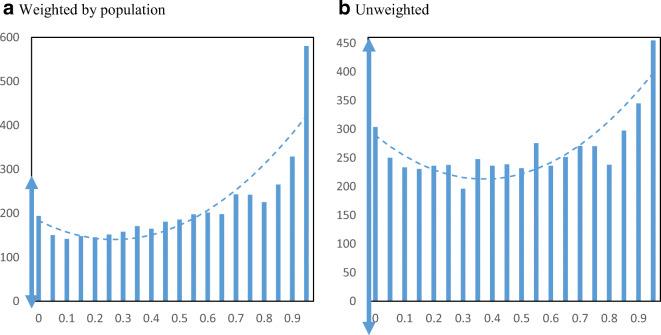
EIDL advance amounts per employee by minority share. Panel **a** Weighted by population. Panel **b** Unweighted. **Notes**: The charts show the mean EIDL advance amounts per employee in zip codes by minority share of the population. The dashed lines are from quadratic regressions at the zip code level. For perspective, the double arrow on the Y-axis indicates the median ± ½ standard deviation

Figure 11 displays EIDL loan amounts per employee by minority share of the zip code. Similar to the number of loans, we find a positive relationship between loan amounts and minority share of the population based on the weighted chart. An increase in EIDL loans per employee from $1404 to $1624 is associated with the interquartile range in minority share across zip codes. Figure 12 displays EIDL advances per employee by minority share. We also find a positive relationship for EIDL advances increasing from a weighted average of $148 to $198 per employee when moving from the 25th to the 75th percentile in minority share.

## Conclusions

Given the shutdown of the economy to slow down the spread of the novel coronavirus, Congress agreed to a massive level of expenditures in the 2020 CARES Act to help small businesses stay open and retain employees. Two components directly providing loan and grant assistance to small businesses, the PPP and EIDL programs, provided a total of nearly 15 million separate loans or advances, and a staggering level of expenditures of roughly $850 billion. The total number and amount of support for small businesses in the USA is unprecedented. Given that the programs were to help disadvantaged businesses (U.S. Congress 2020), we provide the first study of whether loans and advances from these programs were indeed distributed positively to minority communities.

Using administrative data on the universe of 2020 PPP loans, EIDL loans, and EIDL advances, we explore how loans and advances were distributed. We find that funding per business and employee from these programs both flowed to minority communities and away from minority communities. Focusing first on PPP loans, we generally find a slightly positive relationship between PPP loan receipt per business and the minority share of the population. There is some evidence, however, that the first round of funds was disproportionately disbursed to non-minority communities and the second round of funds was disproportionately disbursed to minority communities. When we focus on the minority share of employer businesses in an area, we find similar results: slightly positive relationship but differential relationships by disbursement rounds. Focusing on PPP loan amount per employee, we find a negative relationship with minority share of the population. EIDL loans and advances, in both number per business and amounts per employee, were provided positively to minority communities. We find a strong positive relationship in the receipt per business of these loans and advances by the minority share of the population.

Although analyzing patterns of PPP and EIDL funding receipt per business across minority communities by using the universe of loan-level data across minority communities is important, the loan-level data are limited by not having information on loan receipt by race and ethnicity. To be sure, there is some information in the PPP loan data, but only 10% of loans include race and ethnicity (and in a non-representative way by lender), and none of the loans in the EIDL data provides information on race and ethnicity. There is always the possibility that minority businesses did not evenly receive loans in geographical areas even with high minority shares of the population or high minority shares of businesses. The federal government has been criticized heavily for not collecting this information and plans on collecting demographic information when processing forgiveness on the PPP loans. Future research needs to address this critical question.

Another criticism of the programs is that there was no collection of information on applications for loans that were denied. There is no way to gauge demand and unmet need for these loans by minority businesses and in minority communities. Although there is currently no information by race, the Census Bureau’s Small Business Pulse Survey indicates that by early August, most businesses in their survey who asked for PPP or EIDL funds reported receiving them (US Census Bureau, 2020). But, this is an important concern. There might exist large disparities by race, and there is a major difference in potential policy response between whether minority businesses needed these loans but faced barriers (e.g., lack of established bank relationships, lack of information about loans, digital divide, or discrimination) or if they did not need loans or needed smaller loans. Another concern is that many minority businesses did not have employees and the programs were primarily focused on serving employer businesses. Finally, many minority businesses might have been reluctant to apply for PPP loans because of uncertainty over future revenues due to entering the pandemic in a weakened position (Mills & Battisto, 2020).

The findings presented in this research note have implications for trends in broader inequality. Minority-owned businesses are important for local job creation (as minority owners disproportionately hire minority workers), economic advancement, and longer-term wealth inequality (Boston, 1999, 2006; Bradford, 2003, 2014; Fairlie & Robb, 2008; Stoll et al., 2001). With major losses in business activity among minority businesses in the early stages of the pandemic (Fairlie, 2020), minority business owners have already lost substantial amounts of income from their businesses. The long-term economic consequences on minority businesses could be severe. Many minority business owners do not have the resources to weather prolonged closures, reduced demand from health concerns, and a more comprehensive recession. Just prior to the pandemic when small business owners were asked what actions they would take if faced with a 2-month revenue loss, roughly half said they would use their own funds and 17% said they would close or sell the business (Mills et al., 2020). But, the latest Census data indicate that the median level of wealth among black families is $9600 and Latinx families is $25,000 compared with $172,000 among white families possibly making it difficult to use their own funds for an extended period of time (U.S. Census Bureau, 2017).

The finding that PPP loans reached minority communities better in the second than in the first round of the program suggests that besides making funds available, it also matters how the program is implemented, how and how fast information is conveyed, and through which routes support is offered. In light of the Black Lives Matter movement, there has been a push to support black-owned businesses around the country. In the Economic Aid Act that instantiated a new round of the PPP program on December 27, 2020, Congress set aside funds for community financial institutions and credit unions, and the SBA provided a head start for community financial institutions, among other measures, intending to increase access to PPP loans for small and underserved businesses, including minority businesses. In February 2021, the federal government aimed to increase equitable access further by establishing a 14-day exclusive PPP loan application period for businesses with fewer than 20 employees, and the SBA has reached out by organized informational webinars, including some in the context of Black History Month. Further research is needed to assess the effectiveness of these and other measures to increase equitable access to small business support programs.

## Footnotes

1 These findings prompted a large policy response, for example, new US Senate bills (US Senate, 2020) and California State bills (Newsom, 2020) to provide assistance to minority and small businesses during the pandemic.

2 Cash balances and revenues of small businesses also fell substantially in the early stages of the pandemic (Farrell et al., 2020).

3 The Coronavirus Aid, Relief, and Economic Security (CARES) Act also provided stimulus payments to households and expanded unemployment insurance benefits to households. See Bhutta et al. (2020) for an analysis of whether this cash assistance will help families cover expenses over a 6-month period of lost income (i.e., April through September 2020), relative to a counterfactual where families would have had to rely solely on their own liquid savings and standard UI benefits (e.g., benefits available in the absence of CARES).

4 On March 11, 2020, the World Health Organization (WHO) declared COVID-19 a pandemic. On March 16, the San Francisco Bay Area imposed the first shelter-in-place restrictions in the country followed by the State of California on March 19. New York State followed the next day. By early April, most states imposed social distancing restrictions.

5 Alstadsaeter et al. (2020) use Norwegian administrative data to simulate the effects of Norwegian government support and the US PPP program to help businesses during the COVID-19 pandemic and find that these policies supporting payroll can be partly effective.

6 A large literature explores the causes and consequences of disparities in ownership and success of minority-owned businesses. For broader discussions and reviews of these literature, see, for example, Dávila and Mora (2013); Fairlie and Robb (2008); Kerr and Kerr (2020); Parker (2018). See Fairlie (2020) and Stanford Latino Entrepreneurship Initiative (2020) for evidence of COVID-19 impacts on minority-owned businesses.

7 The top banks providing PPP loans were Bank of America (7%), JPMorgan Chase (5%), Cross River Bank (4%), Kabbage (4%), and Wells Fargo (4%).

8 We exclude PPP loans to agricultural businesses here due to a lack of data on the minority status of farmers.

9 We also examine the relationship between PPP loans per employer business by minority share of the population at the county level. The results are similar to those at the zip code level.
